# Supplementary material for: Context memory formed in medial prefrontal cortex during infancy enhances learning in adulthood
Source: Nat Commun. 2024 Mar 20;15:2475. doi: 10.1038/s41467-024-46734-6 (PMC10954687; doi:10.1038/s41467-024-46734-6)
Supplement: Supplementary file 1 — Supplementary Information [file 41467_2024_46734_MOESM1_ESM.pdf]

Supplementary Information

Context memory formed in medial prefrontal cortex during infancy enhances learning in adulthood

Maria P. Contreras[1,2, †], Marta Mendez[3], Xia Shan[1,2], Julia Fechner[1,2], Anuck Sawangjit[1], Jan Born[1,4,5* ‡], Marion Inostroza[1‡]

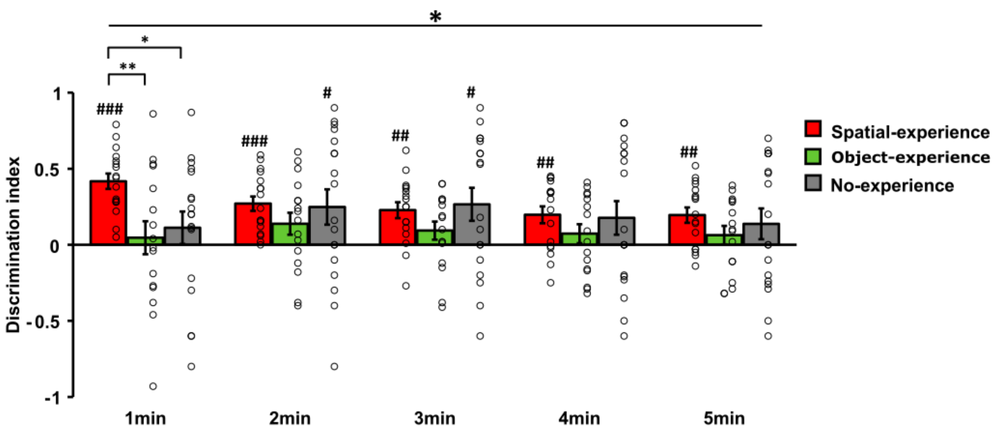

Figure S1. Adult OPR performance for the entire 5-minute retrieval phase. Mean±SEM discrimination ratios separately during the first 1, 2, 3, 4 and entire 5 min of the retrieval phase (dot plots overlaid) at adulthood OPR testing, for the Spatial-experience (red bars, $n = 17$), Object-experience (green bars, $n = 17$), and No-experience (grey bars, $n = 18$) groups. # $P < 0.05$, ## $P < 0.01$ and ### $P < 0.001$, for one-sample t-test against chance level; * $P < 0.05$ and ** $P < 0.01$ for pairwise comparisons (two-sided t-tests) between experimental groups. Horizontal line: $P < 0.05$ for Group x Minute ANOVA interaction. Note, enhanced OPR memory for the Spatial-experience group with, greatest difference between groups in the first min. Source data are provided as a Source Data file.

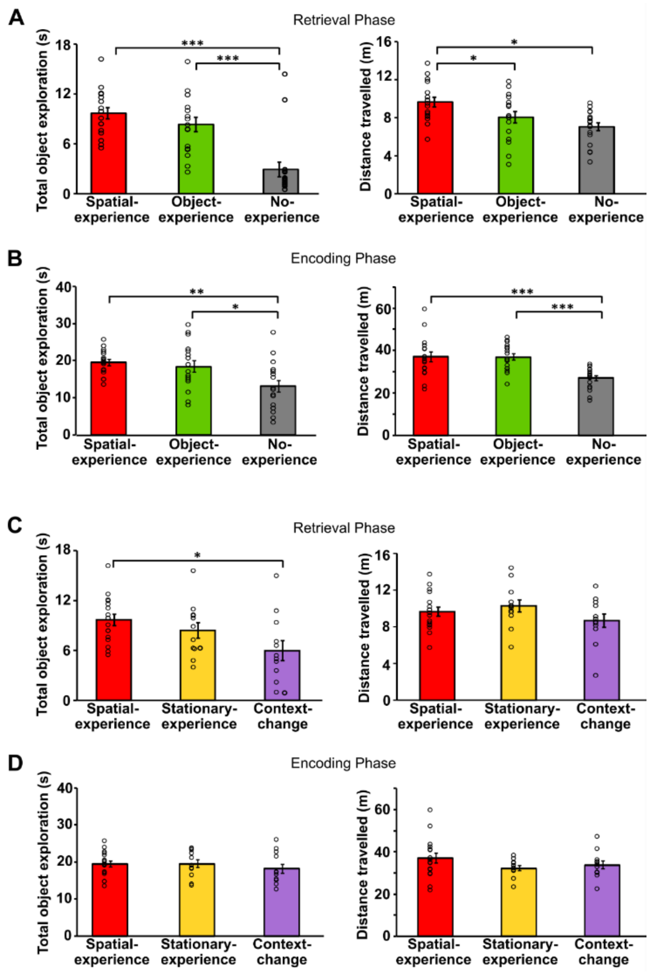

Figure S2. Total exploration time and distance travelled during OPR performance. (A) Total object exploration time (in s) towards both objects (left) and total distance travelled (in m) during the retrieval phase (1st minute) for the Spatial-experience (red bars, n = 17), Object-experience (green bars, n = 17) and No-experience groups (grey bars, n = 18). **(B)** The same control variables for the 5-min encoding phase in these groups. **(C)** Total object exploration time and total distance travelled for the control experiments comparing the Spatial-experience group (red bars) with the Stationary-experience (n = 12 rats, yellow) and the Context-change group (n = 12 rats, purple). **(D)** The same control variables for the 5-min encoding phase in these groups. Means (± SEM) are indicated. * P < 0.05 and *** P < 0.001 for pairwise t-test (two-sided). Note, in (A) Spatial-experience and Object-experience groups were closely comparable with respect to total exploration time. However, the Spatial-experience group travelled a slightly greater distance than the Object-experience group ($t(32)$ = -2.055, P = 0.048), possibly reflecting general arousing effects on locomotion resulting from stimulation specifically of spatial systems during infancy. In (B), both Spatial-experience and Object-experience groups showed increased total object exploration and distance travelled in the encoding phase in comparison with the No-experience control group ($t(33)$ > 3.6, P > 0.001), suggesting an unspecific effect of infantile experience

(independent of the kind of experience). To further exclude any confounding influence of these control variables that differed between groups on OPR retrieval performance, we ran additional ANOVA including these variables as covariate (i.e., distance travelled at retrieval or encoding, and total exploration at encoding). These analyses confirmed the significant differences in the discrimination index between groups (as reported in the main text) in all cases (all $P < 0.034$), with none of the covariates reaching significance (all $P > 0.153$). In (D) the Context-change group showed reduced total exploration time for the objects (when compared to the Spatial-experience group, $t(27) = 2.864$, $P = 0.015$) during retrieval, possibly due to increased context exploration. Additional ANOVA including total exploration time as covariate confirmed a significantly higher discrimination index in the Spatial-experience than the Context-change group ($P = 0.034$) thereby excluding that the differences in OPR memory were confounded by total exploration time ($P = 0.409$ for the covariate). Source data are provided as a Source Data file

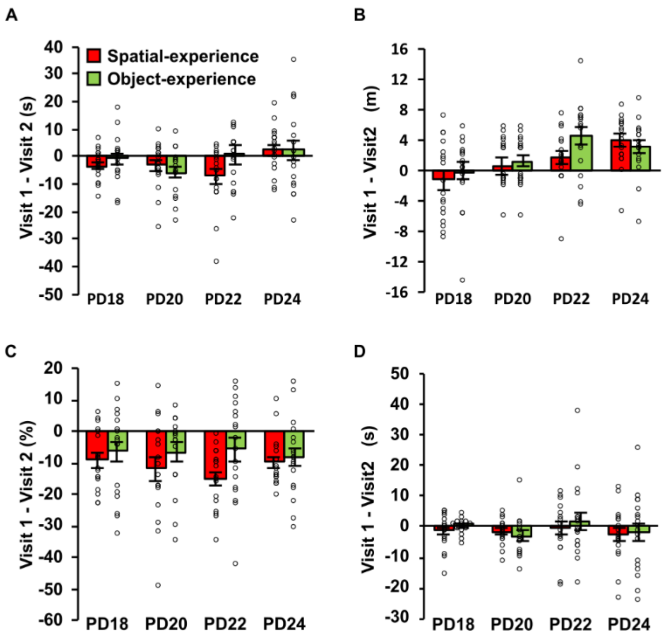

Figure S3. Behavioral parameters for experimental exposures during infancy. For an analysis of signs of the pups' interest in the objects and arena environment at the four exposures during infancy, responses to the change in the spatial configuration and in the objects, respectively, were assessed, separately for each exposure, for **(A)** the Total object exploration (in s), **(B)** Distance travelled (in m), **(C)** Percentage of time spent in the center of the arena (as a proxy of the pup's anxiety levels), and **(D)** Rearing behavior (in s, as an indicator of exploration of distal cues in the arena environment). Means (±SEM) are indicated. For each exposure data are expressed as difference between visit1- visit2. There were no significant differences between the Spatial and Object-experience groups (*n* = 17 for each group, all *P* > 0.157). Source data are provided as a Source Data file

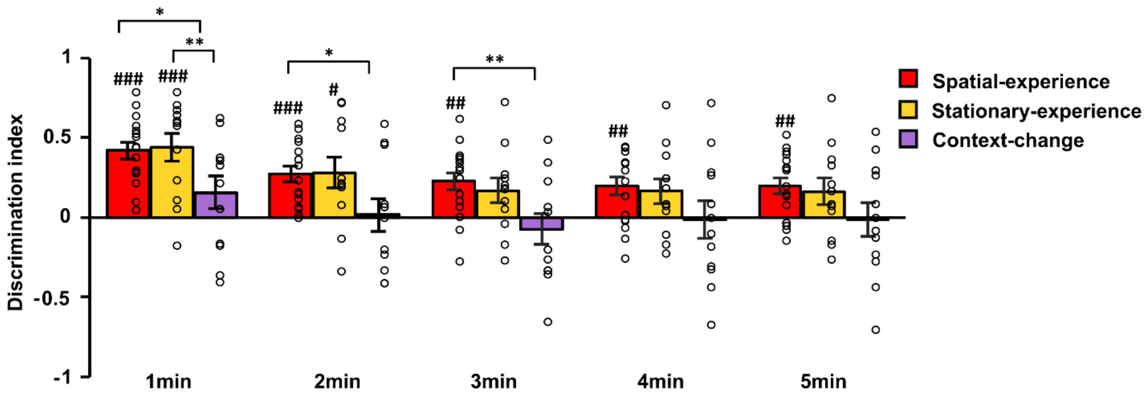

Figure S4. OPR memory for the entire 5-minute retrieval phase in the Stationary-experience and Context-change groups. Mean ± SEM discrimination ratios for the Stationary-experience (*n* = 12 rats, yellow bars) and the Context-change (*n* = 12 rats, purple bars) control groups shown separately during the first 1, 2, 3, 4 and entire 5 min of the retrieval phase, in comparison with the Spatial-experience group (*n* = 17; red bars; dot plots overlaid). For the Stationary-experience group, objects and their spatial configuration remained unchanged at the two visits of each infantile exposure. Procedures for the Context-change group were the same as for the Spatial-experience group, except that OPR testing at adulthood was performed in an entirely different context. ## $P < 0.01$ and ### $P < 0.001$ for one-sample *t*-test against chance level. * $P < 0.05$, and ** $P < 0.01$ for pairwise comparisons (two-sided t-tests) between experimental groups. ($F(1, 27) = 0.064$, $P = 0.803$ and $F(1, 27) = 31.943$, $P = 0.001$, for group main effect in ANOVA comparing the Spatial-experience group with the Stationary-experience and Context-change groups, respectively). Source data are provided as a Source Data file.

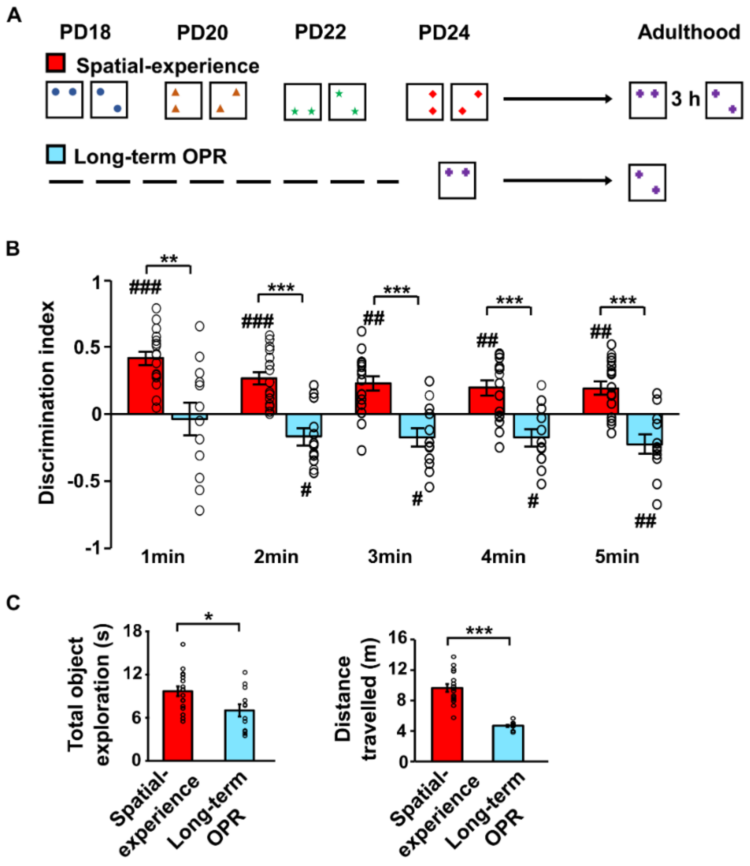

Figure S5. OPR memory for the Long-term OPR control group. Findings are shown in comparison with the Spatial-experience group of the main experiments. **(A)** General procedure for both groups. For the Long-term OPR (*n* = 12 rats, cyan), the OPR encoding phase took place during infancy (PD24) and retrieval testing at adulthood (PD84). **(B)** Mean ± SEM discrimination ratios shown separately for the first 1, 2, 3, 4 and entire 5 min of the retrieval phase, and (**C**) total object exploration time (in s; left) and total distance travelled (in m) during the 1st min of retrieval phase for the Long-term OPR (cyan bars) and Spatial-experience groups (red bars; dot plots overlaid) # $P < 0.05$, ## $P < 0.01$ and ### $P < 0.001$ for one-sample *t*-test against chance level. * $P < 0.05$, ** $P < 0.01$ and *** $P < 0.001$, for pairwise comparisons (two-sided *t*-tests) between experimental groups. In (B), discrimination ratios differed between groups across the entire 5-min phase, $F(1, 27) = 7.490$, $P = 0.011$, for group main effect. Note, starting from the 2nd minute, the Long-term OPR group displayed significant negative discrimination ratios suggesting the presence of a rudimentary form of a memory for the original infantile experience which, curiously, is expressed in an "infantile" manner, namely as familiarity preference (for the stationary object) rather than as novelty preference[1]. In (C), the diminished total exploration time and distance travelled in the Long-term OPR group partly reflect the use of a smaller arena for retrieval testing (because for this group, the encoding phase took place during infancy where a smaller arena was used). The difference in OPR memory between the groups was confirmed in an analysis of covariance on the discrimination index using total exploration time and distance travelled as

covariates ($F(1, 28) = 4.570$, $P = 0.042$ and $F(1,28) = 11.114$, $P = 0.003$, respectively). Source data are provided as a Source Data file.

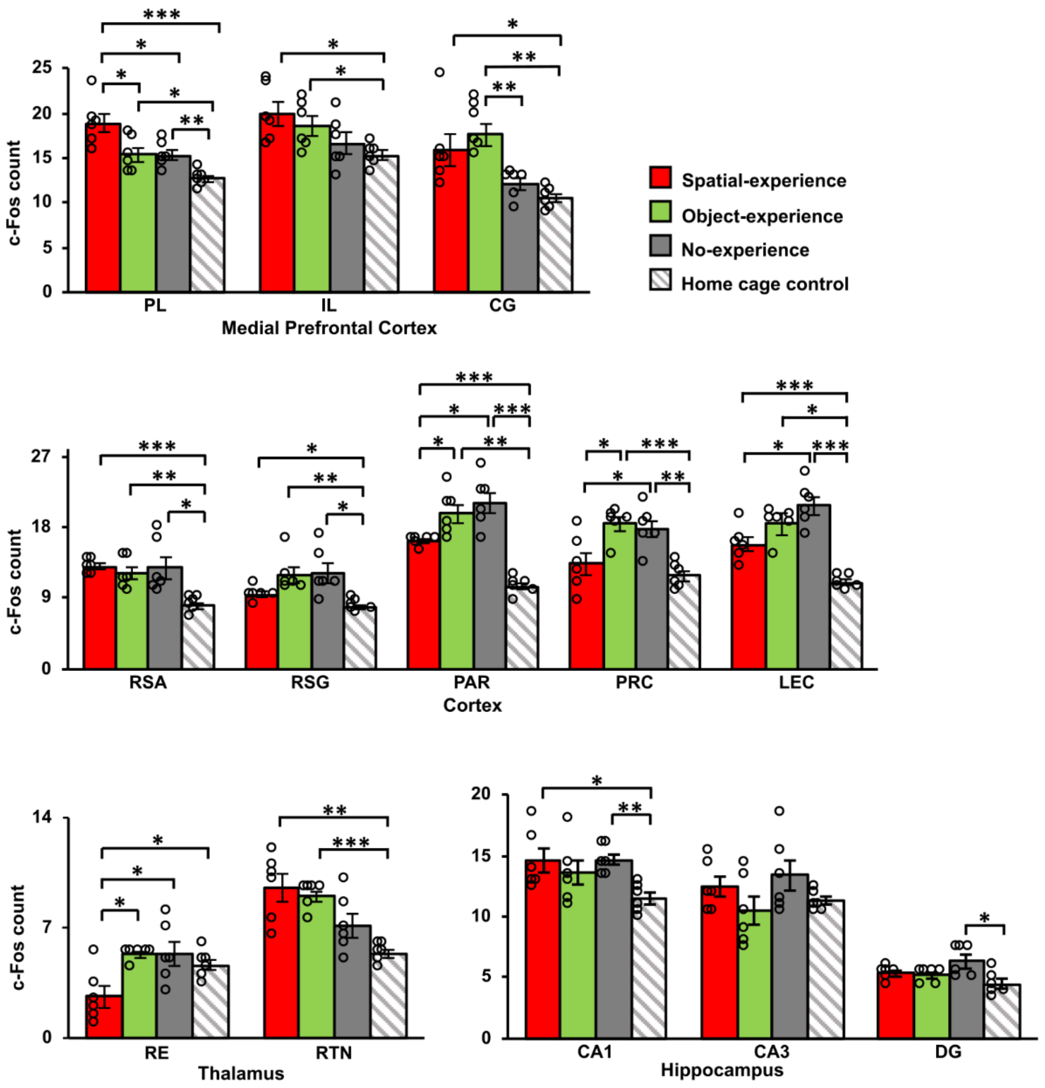

Figure S6. c-Fos activity for all regions of interest in comparisons with a home cage control group. Mean±SEM counts of c-Fos$^+$ cells in the Spatial-experience (red bars, n = 6), Object-experience (green bars, n = 6), No-experience (grey bars, n = 6), and Home cage control groups (hatched bars, n = 6) in (top) subregions of the medial prefrontal cortex, PL - prelimbic cortex, IL - infralimbic cortex, CG - cingulate cortex, (middle) the agranular retrosplenial (RSA), granular retrosplenial (RSG), parietal (PAR), perirhinal (PRC) and lateral entorhinal (LEC) cortices, (bottom left) in thalamic nuclei, RE - nucleus reuniens, RTN - reticular thalamic nucleus, and (bottom right) in hippocampal subfields, CA1 - cornu ammonis 1, CA3 - cornu ammonis 3, DG - dentate gyrus. Home cage control rats (n = 6) remained in their home cage during OPR testing at adulthood but otherwise were subjected to the same experimental procedures (during infancy) as the Spatial-experience group. * $P < 0.05$, ** $P < 0.01$ and *** $P < 0.001$ for pairwise comparisons (two-sided t-test) between experimental groups. Source data are provided as a Source Data file.

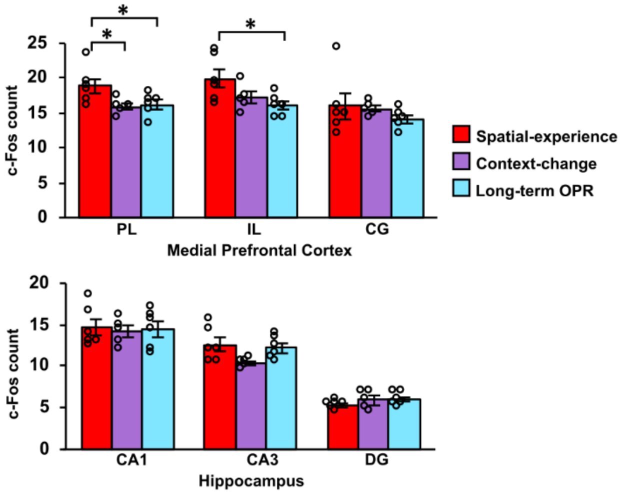

Figure S7. Comparison of c-Fos activity in mPFC and hippocampal areas between the Spatial-experience, Context-change, and Long-term OPR groups. Mean±SEM counts of c-Fos^{+} cells in the Spatial-experience (red bars, n = 6), Context-change groups (purple bars, n = 5), and Long-term OPR (cyan bars, n = 6), in (upper panels) subregions of the medial prefrontal cortex, PL - prelimbic cortex, IL - infralimbic cortex, CG - cingulate cortex, and (lower panels) hippocampal subfields, CA1 - cornu ammonis 1, CA3 - cornu ammonis 3, DG - dentate gyrus. * $P \leq 0.05$, ** $P < 0.01$, and *** $P < 0.001$ for pairwise comparisons (t-test) between experimental groups. In all groups, c-Fos was determined 90 min after the retrieval phase. Note, in comparison with both Long-term OPR and Context-change groups, the Spatial-experience group shows enhanced c-Fos activity in the PL region of the mPFC with no differences in hippocampal regions between groups. Source data are provided as a Source Data file.

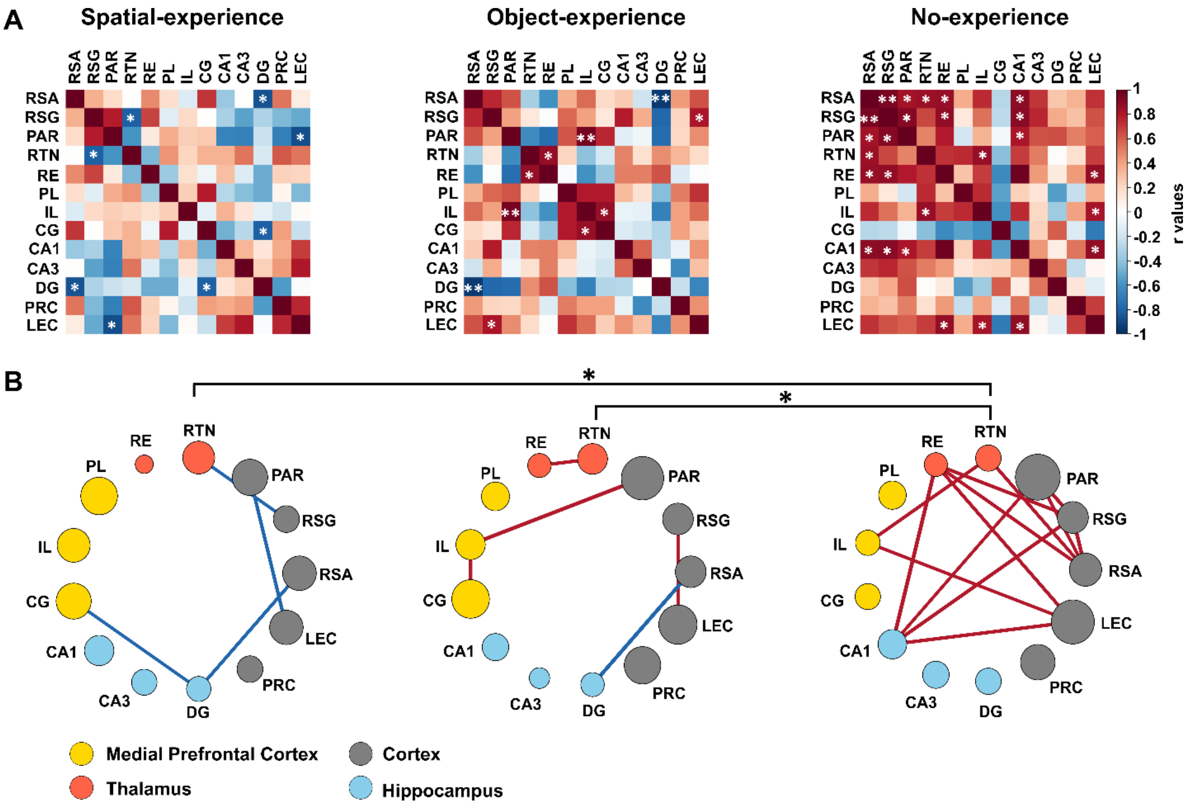

Figure S8. Network functional connectivity analysis based on c-Fos activity at OPR retrieval testing. (A) Pearson correlation matrices showing inter-regional correlations for c-Fos activation in the Spatial-experience, Object-experience, and No-experience groups (each group, $n = 6$). Axes represent the different brain areas. Correlation coefficients are color-coded (scale bars, right) and white asterisk (in the square) indicates significance (*$P < 0.05$ and ** $P < 0.01$, uncorrected for multiple comparisons). **(B)** Network connectivity graphs depicting only correlation coefficients exceeding the criterion size of $r = \pm 0.815$ (corresponding to an uncorrected significance of $P < 0.05$, red: positive, blue: negative). Different brain areas are color-grouped and node size for the Spatial-experience, Object-experience and No-experience groups is proportional to the differential activation in comparison with the Home cage control group (* $P < 0.05$, for the difference in number of significant correlations between groups, for pairwise Fisher's exact test). Note, decrease in the number of significant inter-regional coactivations in the Spatial-experience and Object-experience groups, in comparison with the No-experience control group, with this decorrelation reflecting a non-specific effect of infantile experience producing a general sharpening of arena-related representations that might more effectively process relevant information[2, 3]. Source data are provided as a Source Data file.

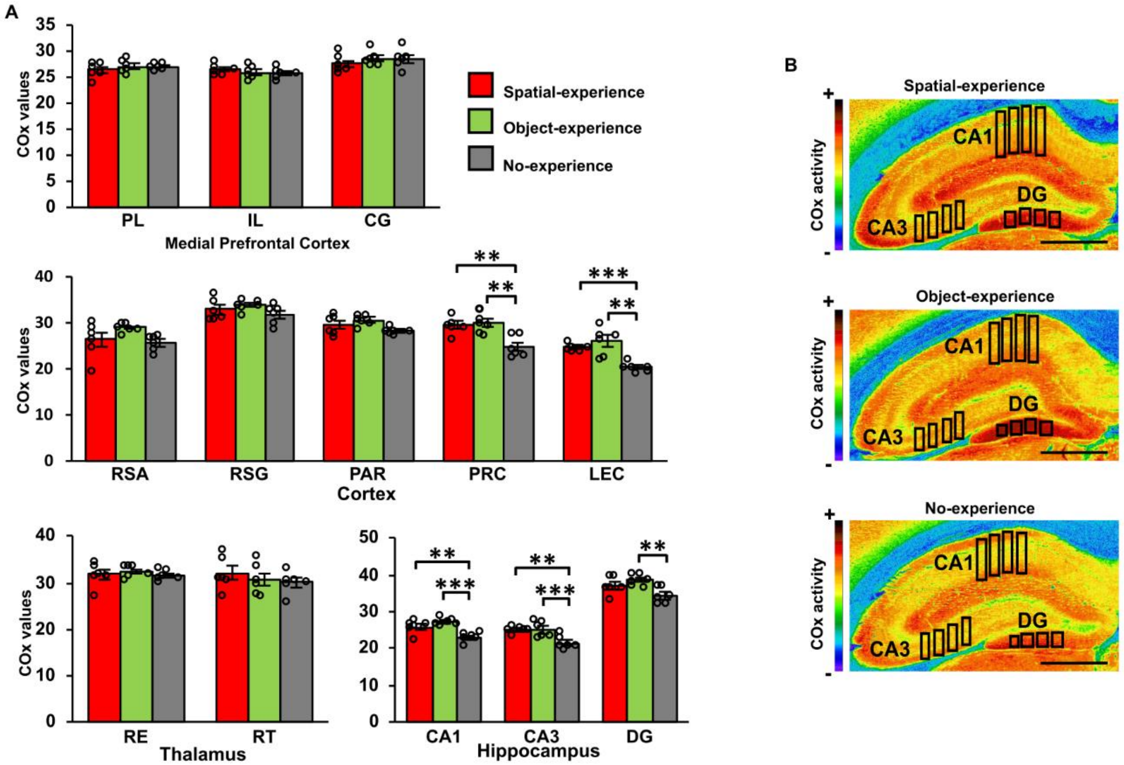

Figure S9. COx activity in cortical, thalamic and hippocampal regions of interest. (A) Mean±SEM values of COx activity in the Spatial-experience (red bars, $n = 6$), Object-experience (green bars, $n = 6$), and No-experience groups (grey bars, $n = 6$) in (top) subregions of the medial prefrontal cortex, PL - prelimbic cortex, IL - infralimbic cortex, CG - cingulate cortex, (middle) the agranular retrosplenial (RSA), granular retrosplenial (RSG), parietal (PAR), perirhinal (PRC) and lateral entorhinal (LEC) cortices, (bottom left) in thalamic nuclei, RE - nucleus reuniens, RTN - reticular thalamic nucleus, and (bottom right) in hippocampal subfields, CA1 - cornu ammonis 1, CA3 - cornu ammonis 3, DG - dentate gyrus. ** $P < 0.01$ and *** $P < 0.001$ for pairwise *t*-test (two-sided). **(B)** Representative images of COx-staining from hippocampal CA1, CA3 and DG regions. Rectangles indicate measured areas (scale bar: 1 mm). COx activity reflects the cell's basic metabolic rate as a trait marker of its activity level[4] and was measured based on optical density estimates (see Methods). Groups with experience (spatial and object) during infancy showed an increase in COx activity in the PRC ($F(2, 17) = 10.126$, $P = 0.002$) and LEC ($F(2, 17) = 14.780$, $P = 0.001$) cortices as well as in the hippocampal CA1 ($F(2, 17) = 12.440$, $P = 0.001$), CA3 ($F(2, 17) = 8.018$, $P = 0.004$) and DG subfields ($F(2, 17) = 5.911$, $P = 0.013$; $F(17.976, 134.819) = 2.428$, $P = 0.002$ for global Group x Area interaction).

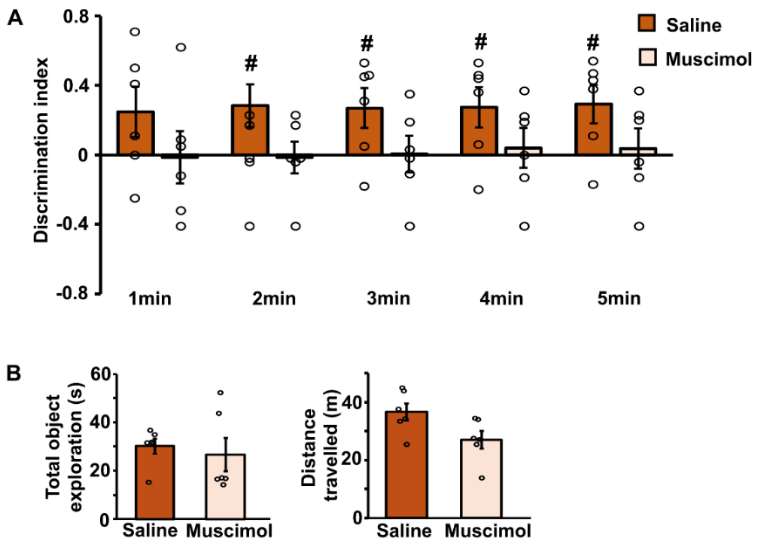

Figure S10. Adult OPR performance for the entire 5-minute retrieval phase for the Saline and Muscimol groups. (A) Mean±SEM discrimination ratios separately during the first 1, 2, 3, 4 and entire 5 min of the retrieval phase at adulthood OPR testing, for the Saline (n = 6) and Muscimol (n = 6) groups (dot plots overlaid). # $P < 0.05$, one sample t-test (two-sided) against chance level. Differences between the groups for the first 3 min were significant ($F(1, 34) = 8.4$, $P < 0.01$, for main effect of Group, $P = 0.84$ for Group x Minutes interaction, see main text). **(B)** Mean±SEM total object exploration time (in s, left) and distance travelled (in m, right) during the 5-min encoding phase. There were no significant differences between conditions (all $P > 0.05$). Source data are provided as a Source Data file.

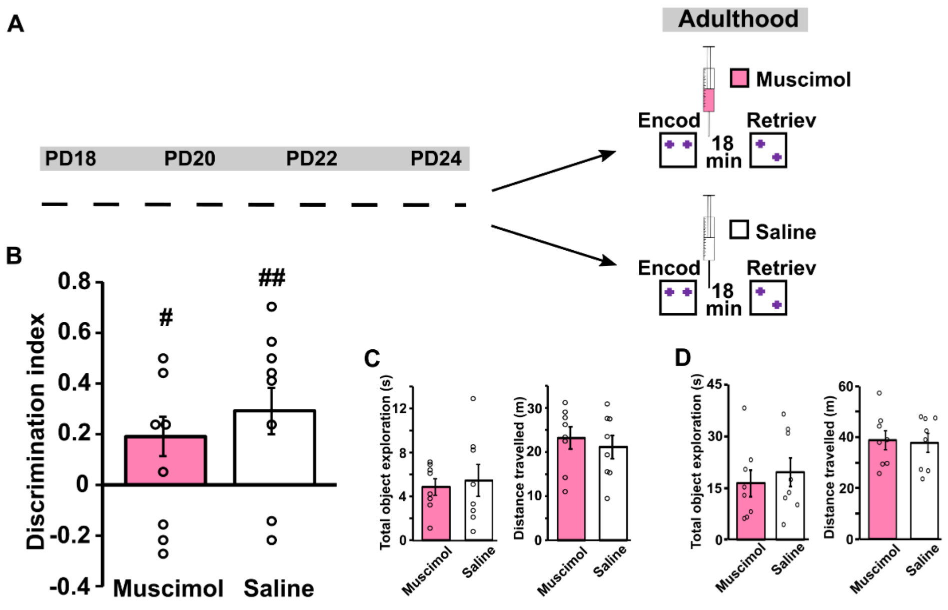

Figure S11. Inhibition of the PL mPFC by muscimol does not impair adult rat's capability for OPR memory. **(A)** Procedures were the same as in the main experiments (Fig. 4) except that the retention interval between encoding and retrieval was shortened to ~18 min, and there was no prior infantile experience. Muscimol/saline was infused immediately after the encoding phase bilaterally over 3 min and 15 min later, the OPR retrieval phase started. Each of 4 rats was tested twice on the muscimol and vehicle conditions, respectively, in random order (using different objects and configurations at each test and a >2 days interval between conditions). (**B**) Discrimination ratios. Mean ± SEM values across first 3 min of retrieval phase are shown, dot plots overlaid (n = 8). **(C)** Total object exploration time (in s, left) and distance travelled (in m, right) during retrieval and **(D)** the 5-min encoding phase. # $P < 0.05$, ## $P < 0.01$, for one-sample t-test (two-sided) against chance level. Note, significant OPR memory in both muscimol and vehicle conditions. There were no significant differences between conditions (all $P > 0.41$). Source data are provided as a Source Data file.

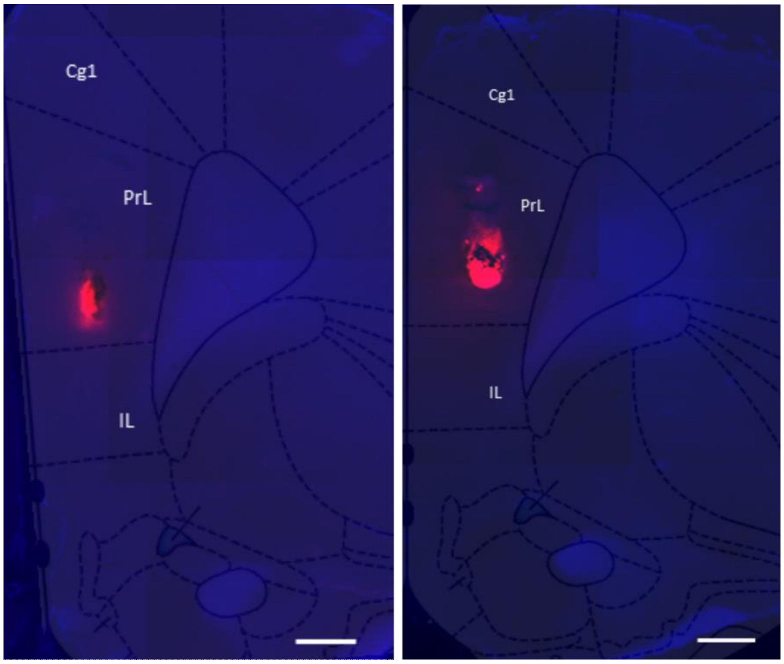

Figure S12. Spreading of fluorescent muscimol in the prelimbic region (PL) of the mPFC. Two representative coronal brain section at 3.24 mm from bregma showing spread of muscimol (red) after infusion into the PL. Sections were visualized under fluorescent microscopy for fluorescent muscimol (0.3 µg dissolved in 0.3 µL of 0.9% saline solution, per hemisphere). Animals were sacrificed 2 days after the infusion and brains were post-fixed with PFA 4% for 24 h. Brains were cut on a vibratome to obtain 70 µm thick sections and stained with DAPI (1:5,000 µl in PBS) for 15 min. Fluorescent images were acquired by epifluorescence microscopy (Axio imager Zeiss, Germany). Scale bar indicates 500 µm.

References

1. Contreras, M.P., Born, J., Inostroza, M. The expression of allocentric object-place recognition memory during development. *Behav. Brain Res*. **372**, 112013 (2019).
2. Frostig, R.D., Functional organization and plasticity in the adult rat barrel cortex: moving out-of-the-box. *Curr. Opin. Neurobiol.* **16**, 445-450 (2006).
3. Averbeck, B.B., Latham, P.E., Pouget, A., Neural correlations, population coding and computation. *Nat. Rev. Neurosci.* **7**, 358-366 (2006).
4. M.T. Wong-Riley, Cytochrome oxidase: an endogenous metabolic marker for neuronal activity. *Trends Neurosci*. **12**, 94-101 (1989).
