# Supplementary material for: Context memory formed in medial prefrontal cortex during infancy enhances learning in adulthood
Source: Nat Commun. 2024 Mar 20;15:2475. doi: 10.1038/s41467-024-46734-6 (PMC10954687; doi:10.1038/s41467-024-46734-6)
Supplement: Supplementary file 3 — Reporting Summary [file 41467_2024_46734_MOESM3_ESM.pdf]

nature portfolio

Corresponding author(s): Jan Born

Last updated by author(s): 26/2/23

Reporting Summary

Nature Portfolio wishes to improve the reproducibility of the work that we publish. This form provides structure for consistency and transparency in reporting. For further information on Nature Portfolio policies, see our Editorial Policies and the Editorial Policy Checklist.

Statistics

For all statistical analyses, confirm that the following items are present in the figure legend, table legend, main text, or Methods section.

n/a	Confirmed	
☐	☒	The exact sample size (*n*) for each experimental group/condition, given as a discrete number and unit of measurement
☐	☒	A statement on whether measurements were taken from distinct samples or whether the same sample was measured repeatedly
☐	☒	The statistical test(s) used AND whether they are one- or two-sided<br>*Only common tests should be described solely by name; describe more complex techniques in the Methods section.*
☐	☒	A description of all covariates tested
☐	☒	A description of any assumptions or corrections, such as tests of normality and adjustment for multiple comparisons
☐	☒	A full description of the statistical parameters including central tendency (e.g. means) or other basic estimates (e.g. regression coefficient) AND variation (e.g. standard deviation) or associated estimates of uncertainty (e.g. confidence intervals)
☐	☒	For null hypothesis testing, the test statistic (e.g. *F*, *t*, *r*) with confidence intervals, effect sizes, degrees of freedom and *P* value noted<br>*Give P values as exact values whenever suitable.*
☒	☐	For Bayesian analysis, information on the choice of priors and Markov chain Monte Carlo settings
☒	☐	For hierarchical and complex designs, identification of the appropriate level for tests and full reporting of outcomes
☐	☒	Estimates of effect sizes (e.g. Cohen's *d*, Pearson's *r*), indicating how they were calculated

Our web collection on statistics for biologists contains articles on many of the points above.

Software and code

Policy information about availability of computer code

Data collection	The rat’s behavior was video-recorded during the visits of the early experience as well as during the encoding and retrieval phases of the OPR task and visually scored offline by an experienced experimenter using the ANY-Maze tracking software (Stoelting Europe, Dublin, Ireland).
Data analysis	All statistical analyses were performed using SPSS software (IBM, Armonk, NY, USA). For connectivity graphs, the Igraph package (v1.2.4.2) in R (RStudio, Boston, MA) was used.

For manuscripts utilizing custom algorithms or software that are central to the research but not yet described in published literature, software must be made available to editors and reviewers. We strongly encourage code deposition in a community repository (e.g. GitHub). See the Nature Portfolio guidelines for submitting code & software for further information.

Data

Policy information about availability of data

All manuscripts must include a data availability statement. This statement should provide the following information, where applicable:

- Accession codes, unique identifiers, or web links for publicly available datasets
- A description of any restrictions on data availability
- For clinical datasets or third party data, please ensure that the statement adheres to our policy

All data needed to evaluate the conclusions in the paper are present in the paper and/or the Supplementary Material/Source Data. Source data are provided with this paper.

Research involving human participants, their data, or biological material

Policy information about studies with human participants or human data. See also policy information about sex, gender (identity/presentation), and sexual orientation and race, ethnicity and racism.

Reporting on sex and gender	n/a
Reporting on race, ethnicity, or other socially relevant groupings	n/a
Population characteristics	n/a
Recruitment	n/a
Ethics oversight	n/a

Note that full information on the approval of the study protocol must also be provided in the manuscript.

Field-specific reporting

Please select the one below that is the best fit for your research. If you are not sure, read the appropriate sections before making your selection.

☒ Life sciences ☐ Behavioural & social sciences ☐ Ecological, evolutionary & environmental sciences

For a reference copy of the document with all sections, see nature.com/documents/nr-reporting-summary-flat.pdf

Life sciences study design

All studies must disclose on these points even when the disclosure is negative.

Sample size	Sample sizes were determined based on similar studies in the field of memory consolidation and memory development carried out by our group (Sawangjit, A., Oyanedel, C. N., Niethard, N., Salazar, C., Born, J., & Inostroza, M. (2018). The hippocampus is crucial for forming non-hippocampal long-term memory during sleep. Nature, 564(7734), 109–113; Contreras, M. P., Born, J., & Inostroza, M. (2019). The expression of allocentric object-place recognition memory during development. Behavioural brain research, 372, 112013.) or from other groups (e.g., Travaglia, A., Bisaz, R., Sweet, E. S., Blitzer, R. D., & Alberini, C. M. (2016). Infantile amnesia reflects a developmental critical period for hippocampal learning. Nature neuroscience, 19(9), 1225–1233).
Data exclusions	Statistical outliers were defined by a DI in the 1st min of the retrieval phase exceeding ±1.5 times the interquartile range (which correspond to the difference between the first and third quartile) were excluded from analyses (1 case each in the Spatial-experience and Object-experience group, 2 cases in the Spatial-experience replication group). This exclusion criteria was defined a priori in accordance with previous publications from our group (e.g., Contreras, M. P., Born, J., & Inostroza, M. (2019). The expression of allocentric object-place recognition memory during development. Behavioural brain research, 372, 112013.; Contreras, M. P., Fechner, J., Born, J., & Inostroza, M. (2023). Accelerating Maturation of Spatial Memory Systems by Experience: Evidence from Sleep Oscillation Signatures of Memory Processing. The Journal of neuroscience: J Neurosci. 43(19), 3509–3519). Two rats of the prelimbic inactivation experiments were excluded from the analyses because the correct placement of the cannula could not be confirmed (from reference line 696-697 in the updated manuscript file).
Replication	The Spatial-experience and No-experience groups (shown in Figure 1B) were replicated and pooled together with the respective groups of the main experiments after confirming that (i) the target effect of an enhanced OPR memory was replicated in these animals ($F(1, 20) = 5.437$, $P = 0.030$, for the difference in OPR memory between groups) and (ii) that the groups in OPR memory performance did not differ from the respective groups of the main experiments ($P > 0.642$, for all independent t-test comparisons on each minute of the OPR memory test).
Randomization	Allocation of rats to groups/conditions in all cases was randomized.
Blinding	The experimenter was not blinded to the experimental condition while running the experiments as the condition can be inferred from the experimental procedure. This is because all experimenters were aware of the experimental design. But importantly, the scoring of the animal's behavior (which is the main readout of the present experiment) was performed in a blinded manner with the scorer being unaware of which object was the familiar and the displaced object.

Reporting for specific materials, systems and methods

We require information from authors about some types of materials, experimental systems and methods used in many studies. Here, indicate whether each material, system or method listed is relevant to your study. If you are not sure if a list item applies to your research, read the appropriate section before selecting a response.

Materials & experimental systems

n/a	Involved in the study
☐	☒ Antibodies
☒	☐ Eukaryotic cell lines
☒	☐ Palaeontology and archaeology
☐	☒ Animals and other organisms
☒	☐ Clinical data
☒	☐ Dual use research of concern
☒	☐ Plants

Methods

n/a	Involved in the study
☒	☐ ChIP-seq
☒	☐ Flow cytometry
☒	☐ MRI-based neuroimaging

Antibodies

Antibodies used

For c-Fos staining, rabbit polyclonal anti-c-Fos solution (1:10,000, # sc-52, RRID:AB 2106783, Santa Cruz Biotech, Santa Cruz, CA, USA) and goat anti-rabbit biotinylated IgG secondary antibody (Thermo Scientific Pierce, Rockford, IL, USA; #31820; RRID: AB_228340; diluted 1:200 in incubating solution) were used.

Validation

For: Rabbit polyclonal anti-c-Fos solution (1:10,000, sc-52, Santa Cruz Biotech, Santa Cruz, CA, USA)
Citation: (Santa Cruz Biotechnology Cat# sc-52, RRID:AB 2106783)

https://www.antibodyregistry.org/AB_2106783

Santa Cruz Biotechnology data sheets:
https://datasheets.scbt.com/sc-52.pdf

Name: c-Fos Antibody (4)
ID AB_2106783
Catalog number sc-52
Target antigen: FOS - human, mouse, rat
Clonality: polyclonal
Clone ID 4
Host organism: Rabbit
Provided volumen (100 µg/ml)

Applications: ELISA, Flow Cytometry, Immunocytochemistry, Immunofluorescence, Immunohistochemistry, Immunoprecipitation, Western Blot, Immunohistochemistry(P).

Antibody Profile in CiteAB database:
https://www.citeab.com/antibodies/790361-sc-52-c-fos-antibody-4

The c-Fos (4) has been discontinued and replaced by c-Fos (E-8): sc-166940.

Relevant Citations using this antibody:

Radwanska, K., Medvedev, N. I., Pereira, G. S., Engmann, O., Thiede, N., Moraes, M. F., Villers, A., Irvine, E. E., Maunganidze, N. S., Pyza, E. M., Ris, L., Szymańska, M., Lipiński, M., Kaczmarek, L., Stewart, M. G., & Giese, K. P. (2011). Mechanism for long-term memory formation when synaptic strengthening is impaired. Proceedings of the National Academy of Sciences of the United States of America, 108(45), 18471–18475. https://doi.org/10.1073/pnas.1109680108

Hsieh, H. C., Li, H. Y., Lin, M. Y., Chiou, Y. F., Lin, S. Y., Wong, C. H., & Chen, J. C. (2002). Spatial and temporal profile of haloperidol-induced immediate-early gene expression and phosphoCREB binding in the dorsal and ventral striatum of amphetamine-sensitized rats. Synapse (New York, N.Y.), 45(4), 230–244. https://doi.org/10.1002/syn.10099

El Hage C, Rappeneau V, Etievant A, Morel AL, Scarna H, Zimmer L, Bérod A. Enhanced anxiety observed in cocaine withdrawn rats is associated with altered reactivity of the dorsomedial prefrontal cortex. PLoS One. 2012;7(8):e43535. doi: 10.1371/journal.pone.0043535.

For: Goat anti-rabbit biotinylated IgG secondary antibody (Thermo Scientific Pierce, Rockford, IL, USA; diluted 1:200 in incubating solution)
Citation: (Thermo Fisher Scientific, Cat# 31820; RRID: AB_228340)
https://www.antibodyregistry.org/AB_228340

Thermo Fisher Scientific data sheet:
https://www.thermofisher.com/order/genome-database/dataSheetPdf?productype=antibody&productsubtype=antibody_secondary&productId=31820&version=374

Name Goat anti-Rabbit IgG (H+L) Secondary Antibody, Biotin
ID AB_228340
Catalog number 31820
Target antigen Rabbit IgG (H+L) - rabbit

Clonality: polyclonal secondary
Clone ID N/A
Host organism: Goat

Tissue species: House mouse, Human, Rat, Domestic Dog, others.

Applications: Immunocytochemistry (1:500-1:5,000), Immunohistochemistry (P) (1:500-1:5,000), Western Blot (1:50,000-1:200,000), In Situ Hybridization (Assay-dependent)

Antibody Profile in CiteAB database:
https://www.citeab.com/antibodies/12179342-31820-goat-anti-rabbit-igg-h-l-secondary-antibody?des=08fd6754c24e207c

Relevant Citations using this antibody:

Lyu J, Nagarajan R, Kambali M, Wang M, Rudolph U. Selective inhibition of somatostatin-positive dentate hilar interneurons induces age-related cellular changes and cognitive dysfunction. PNAS Nexus. 2023 Apr 13;2(5):pgad134. doi: 10.1093/pnasnexus/pgad134.

Azevedo-Pereira RL, Manley NC, Dong C, Zhang Y, Lee AG, Zatulovskaia Y, Gupta V, Vu J, Han S, Berry JE, Bliss TM, Steinberg GK. Decoding the molecular crosstalk between grafted stem cells and the stroke-injured brain. Cell Rep. 2023 Apr 25;42(4):112353. doi: 10.1016/j.celrep.2023.112353. Epub 2023 Apr 11. Erratum in: Cell Rep. 2023 Aug 29;42(8):113002.

Animals and other research organisms

Policy information about studies involving animals; ARRIVE guidelines recommended for reporting animal research, and Sex and Gender in Research

Laboratory animals	Experiments were performed on Long-Evans rats from Janvier arrived on post-natal day 8 (Le Genest-Saint-Isle, France), or rats born in our own facilities. Experimental manipulation on these animals started from post-natal day 12 (for groups with infantile experiencees), and manipulation during adulthood occurred on post-natal dat 78-94.
Wild animals	No wild animals were used in the study.
Reporting on sex	Only male rats were used.
Field-collected samples	No field collected samples were used in the study.
Ethics oversight	All experimental procedures were performed in accordance with the European animal protection laws (Directive 2010/63/EU, European Community) and were approved by the Baden-Württemberg state authority.

Note that full information on the approval of the study protocol must also be provided in the manuscript.

Plants

Seed stocks	n.a.
Novel plant genotypes	n.a.
Authentication	n.a.
